# Phagocytosis underpins the biotrophic lifestyle of intracellular parasites in the class Phytomyxea (Rhizaria)

**DOI:** 10.1111/nph.18828

**Published:** 2023-03-24

**Authors:** Andrea Garvetto, Pedro Murúa, Martin Kirchmair, Willibald Salvenmoser, Michaela Hittorf, Stefan Ciaghi, Srilakshmy L. Harikrishnan, Claire M. M. Gachon, John A. Burns, Sigrid Neuhauser

**Affiliations:** ^1^ Institute of Microbiology University of Innsbruck Technikerstraße 25 Innsbruck 6020 Tyrol Austria; ^2^ Laboratorio de Macroalgas, Instituto de Acuicultura Universidad Austral de Chile Puerto Montt 5480000 Chile; ^3^ Institute of Zoology University of Innsbruck Technikerstraße 25 Innsbruck 6020 Tyrol Austria; ^4^ Centre for Plant Systems Biology VIB Zwijnaarde 71 Ghent 9052 Belgium; ^5^ Department of Plant Biotechnology and Bioinformatics Ghent University Zwijnaarde 71 Ghent 9052 Belgium; ^6^ Muséum National d'Histoire Naturelle, UMR 7245, CNRS CP 26 57 rue Cuvier 75005 Paris France; ^7^ Scottish Association for Marine Science Scottish Marine Institute Dunbeg Oban PA37 1QA UK; ^8^ Bigelow Laboratory for Ocean Sciences 60 Bigelow Dr. East Boothbay ME 04544 USA

**Keywords:** algal pathogen, Brassicaceae, *Ectocarpus siliculosus*, *Maullinia ectocarpii*, phagotrophy, plant pathogen, *Plasmodiophora brassicae*, trophic mode

## Abstract

- Phytomyxea are intracellular biotrophic parasites infecting plants and stramenopiles, including the agriculturally impactful *Plasmodiophora brassicae* and the brown seaweed pathogen *Maullinia ectocarpii*. They belong to the clade Rhizaria, where phagotrophy is the main mode of nutrition. Phagocytosis is a complex trait of eukaryotes, well documented for free‐living unicellular eukaryotes and specific cellular types of animals. Data on phagocytosis in intracellular, biotrophic parasites are scant. Phagocytosis, where parts of the host cell are consumed at once, is seemingly at odds with intracellular biotrophy.
- Here we provide evidence that phagotrophy is part of the nutritional strategy of Phytomyxea, using morphological and genetic data (including a novel transcriptome of *M. ectocarpii*). We document intracellular phagocytosis in *P. brassicae* and *M. ectocarpii* by transmission electron microscopy and fluorescent *in situ* hybridization.
- Our investigations confirm molecular signatures of phagocytosis in Phytomyxea and hint at a small specialized subset of genes used for intracellular phagocytosis. Microscopic evidence confirms the existence of intracellular phagocytosis, which in Phytomyxea targets primarily host organelles.
- Phagocytosis seems to coexist with the manipulation of host physiology typical of biotrophic interactions. Our findings resolve long debated questions on the feeding behaviour of Phytomyxea, suggesting an unrecognized role for phagocytosis in biotrophic interactions.

## Introduction

Often seen as a conserved and nearly universal trait, present in nearly all major eukaryote lineages, phagocytosis underpins defining eukaryotic features such as the origin of endosymbiotic organelles and of the endomembrane system (Raven *et al*., 2009; Yutin *et al*., 2009). Phagocytosis is defined as the interiorization and internal digestion of particles larger than 0.5 μm (Flannagan *et al*., 2012) and it is assumed to be the principal mode of nutrition in the majority of free‐living heterotrophic microbial eukaryotes (thereby called phagotrophy). Other heterotrophic microbial eukaryotes feed by osmotrophy (i.e. the extracellular digestion and/or absorption of molecules via the cell membrane) or pinocytosis (i.e. the engulfment of solubilized particles by membrane invaginations), both of which are common in intracellular parasites, though not exclusive to them (Seenivasan *et al*., 2013; Spielmann *et al*., 2020).

Despite its pervasiveness among eukaryotes, we owe most of the information on phagocytosis to a special group of ‘professional phagocytes’ from the immune system of vertebrate model organisms (Uribe‐Querol & Rosales, 2020), for which molecular tools and laboratory experiments are possible. The investigation of phagotrophy and other trophic modes can be challenging in microbial eukaryotes and it relies heavily on microscopic observations and on the labelling/tracking of food items (Keymer *et al*., 2017; Miura *et al*., 2017; Godrijan *et al*., 2022). Even extensive observational evidence may alone be insufficient in establishing the feeding strategy of an organism (Not *et al*., 2007; Moreira & Lopez‐Garcia, 2014). Phagotrophy is used as an example of a range of ‘nearly behavioural’ traits of microbial eukaryotes, the study of which requires a combination of molecular and laboratory‐based investigations (Keeling, 2019).

Intracellular eukaryotic parasites can obtain macromolecules from their host via endocytosis, that is phagocytosis of solid food particles and pinocytosis of fluids and the solutes therein. For example, Apicomplexa such as *Plasmodium* spp. (Abu Bakar *et al*., 2010; Matz *et al*., 2020) and *Toxoplasma gondii* (Dou *et al*., 2014) ingest and digest macromolecules and pieces of host cell cytoplasm via endocytosis. The kinetoplastid, *Trypanosoma cruzii*, has been reported to phagocytotically take up nutrients via the cytostome, a well‐defined tubular structure conserved from its free‐living ancestors (Chasen *et al*., 2020). Some intracellular parasites of fungi, oomycetes and green algae (*Rozella polyphagi* and *Rozella allomycis*; Fungi, Cryptomycota) have been observed to actively engulf host cytoplasm and organelles, but also to recruit host mitochondria around their thallus, seemingly compensating for their own unstructured and depauperated ones (James *et al*., 2013; Powell *et al*., 2017). These findings place *Rozella* (together with the earlier‐cited intracellular parasites of animals) in a particular trophic niche where conserved traits from free‐living ancestors (e.g. phagotrophy) and derived traits co‐evolved with the host (e.g. host manipulation) coexist within the same biotrophic organism.

With the notable exception of the photosynthetic chlorarachnids, phagotrophy is assumed to be the main mode of nutrition in almost all the free‐living Rhizarians (Cavalier‐Smith *et al*., 2018). Within this clade, Phytomyxea are a class of unicellular eukaryotic parasites living as intracellular obligate biotrophs in plants and stramenopiles in marine, freshwater and terrestrial habitats (Bulman & Neuhauser, 2017; Cavalier‐Smith *et al*., 2018). The class is currently split into three main clades: the orders Plasmodiophorida and Phagomyxida (Hittorf *et al*., 2020) and the recently described genus *Marinomyxa* (Kolátková *et al*., 2020). Phylogenetically, Phytomyxea are sister to the free‐living Vampyrellida (Sierra *et al*., 2016; Cavalier‐Smith *et al*., 2018) and Aquavolonida (Bass *et al*., 2018). Aquavolonida are a group of small, unicellular, free‐living phagotrophic flagellates (Bass *et al*., 2018). Vampyrellida are amoebae with different modes of prey item consumption, ranging from classic phagocytic predation to specialized protoplast feeding; in which the target cell wall is perforated and the amoeba infiltrates the space between the wall and the plasma membrane to phagocytize its prey (Hess & Suthaus, 2022). Phytomyxea use a different strategy to achieve a similar result, piercing the cell wall with a sophisticated extrusome called Rohr and Stachel (Keskin & Fuchs, 1969; Aist & Williams, 1971) to gain access to the host cytoplasm (Williams & McNabola, 1970; Maier *et al*., 2000); although clear evidence on how exactly phytomyxids cross the host plasma membrane is still missing. Phytomyxea reach the host cell as flagellated zoospores and penetrate into it as small unicellular protoplasts, later developing into larger intracellular multinucleate feeding plasmodia. Plasmodia can be of two types: short‐lived (*c*. 7 d) sporangial plasmodia, developing into clusters of sporangia (i.e. sporangiosori) and directly releasing infective flagellated zoospores; or sporogenic plasmodia actively growing as biotrophs inside the living host cell (*c*. 3–4 wk) before developing thick‐walled overwintering resting spores (i.e. sporosori). During that time, sporogenic plasmodia induce hypertrophy of the infected cells which, coupled with induced hyperplasia of the tissue, leads to the formation of galls in the host (Murúa *et al*., 2017; Olszak *et al*., 2019). Manipulation of Brassicaceae hosts by *Plasmodiophora brassicae* induces hypertrophied infected cells to act as physiological sinks, driving photosynthates from the aerial parts of the plant (Malinowski *et al*., 2019) and inducing their accumulation as starch grains in the root (Ma *et al*., 2022).

How Phytomyxea feed on their host has never been clearly elucidated and even the trophic mode of the model phytomyxean *P. brassicae* is still debated (Bulman & Neuhauser, 2017). Among the Phagomyxida, the brown seaweed parasite *Phagomyxa algarum* and the diatom parasites *Phagomyxa odontellae* and *Phagomyxa bellerocheae* have been observed to ingest the cytoplasm and organelles from their hosts by phagocytosis and accumulate the digested material in pigmented digestive vacuoles (Karling, 1944; Schnepf, 1994; Schnepf *et al*., 2000). On the other hand, the lack of a conspicuous digestive vacuole and failure to detect engulfed host organelles has led to the conclusion that another brown seaweed‐infecting phagomyxid *Maullinia ectocarpii* feeds by osmotrophy (Maier *et al*., 2000). Within the Plasmodiophorida, intracellular phagotrophy has been observed in the oomycete‐infecting species *Woronina pythii* (Dylewski *et al*., 1978) and *Octomyxa brevilegniae* (Couch *et al*., 1939; Pendergrass, 1950). Food vacuoles containing residues of cytoplasm and organelles from the host plant *Nasturtium officinale* (watercress) have also been found in *Hillenburgia nasturtii* (formerly *Spongospora subterranea* f. sp. *nasturtii*; Clay & Walsh, 1997; Hittorf *et al*., 2020). Despite sparse electron microscopy evidence supporting the existence of phagotrophy in *P. brassicae* (Williams & McNabola, 1967; Buczacki, 1983), a clear consensus on whether nutrition is dominated by osmotrophy, phagotrophy or consists of a mix of the two has not yet been reached (Dylewski, 1990).

Molecularly, complex and ‘behavioural’ traits such as feeding modes are inherently difficult to investigate, since they are the final phenotypic outcome of a cohort of finely tuned genes involved in a range of overlapping (and often widely conserved) biological processes (Keeling, 2019). *In silico* predictions based on the presence or absence of genome‐wide molecular signatures identified in organisms known to possess a certain phenotypic trait can be used to infer the likelihood of the existence of that specific trait in other organisms, based on their genomic information (Burns *et al*., 2018). Direct observation, laboratory‐based experiments and analysis of molecular data are complementary and have been successfully used to identify or rule‐out phagotrophy in different groups of prasinophyte green algae (Bock *et al*., 2021; Jimenez *et al*., 2021).

In this study, we used genomic and transcriptomic data from the plasmodiophorids *P. brassicae* and *S. subterranea* (Schwelm *et al*., 2015; Rolfe *et al*., 2016; Ciaghi *et al*., 2018, 2019); and sequenced the transcriptome of the infective stage of the phagomyxid *M. ectocarpii* to detect molecular signatures of phagotrophic behaviour (i.e. protein families present in well‐known phagocytes) in the class Phytomyxea. We complemented results from these analyses with fluorescent and electron microscopy observations, to investigate whether: intracellular plasmodia engulf organelles and parts of the host cell; the molecular machinery underpinning the phagocytic behaviour is present; and intracellular plasmodia express core genes involved in phagocytosis, similarly to other intracellular phagocytes (e.g. *R. allomycis*).

## Materials and Methods

### *Maullinia ectocarpii* transcriptome: biological material, RNA extraction, sequencing and data processing

The model brown alga *Ectocarpus siliculosus* (Dillwyn) Lyngbye, 1819 strain Ec32m (CCAP 1310/4) was used as a host for the co‐cultivation of *M. ectocarpii* Maier, E. R. Parodi, R. Westermeier et D. G. Müller 2000 (CCAP 1538/1) for RNA extractions. The pathosystem was maintained in half strength Provasoli medium at 15°C, with a 12 h : 12 h photoperiod, and an irradiance of 10 μmol m^−2^ s^−1^. Quadruplicates of *E. siliculosus* Ec32m infected with *M. ectocarpii* were generated, harvested after 21 d with a 70 μm cell strainer (VWR, Radnor, PA, USA), and transferred immediately to ice‐cold RNAlater (Ambion, Austin, TX, USA), stored overnight at 4°C, and transferred at −80°C until used for RNA extraction. Samples in RNAlater were thawed on ice, vortexed and briefly spun down. Five hundred microlitre was transferred onto a pre‐mixed Bead‐matrix (D1034‐MX; Biozym, Hessisch Oldendorf, Germany). Samples were then spun down at 10 000 ***g***, 4°C for 10 min, and RNAlater was carefully removed. Samples were immediately snap frozen in liquid nitrogen. Frozen material was subsequently homogenized with a FastPrep (MP Biomedicals, Santa Ana, CA, USA) for 40 s at 6 m s^−1^. This step was repeated three times and samples were returned into liquid nitrogen in between the three cycles to aid homogenization and avoid RNA degradation. After the last homogenization round, samples were transferred into liquid nitrogen and placed on ice. Four hundred and fifty microlitre buffer RLT (+β‐mercaptoethanol) from the Qiagen RNeasy Plant Mini Kit (Qiagen) were added, samples were vortexed for 30 s and spun down briefly before processing them according to the manufacturer's instructions with an additional ethanol (95%) washing step before RNA elution. RNA quality was tested on an Agilent Bioanalyzer 2100 (Agilent Technologies, Palo Alto, CA, USA). Poly‐A selected strand‐specific library construction and paired‐end sequencing (2× 125 bp on a HiSeq 2500 using v.4 chemistry; Illumina, San Diego, CA, USA) was performed at the VBCF NGS Unit (Vienna, Austria). Quality of the raw reads was checked using Fastqc v.0.9.1 (Andrews, 2010). Illumina adapters were removed and only good quality reads (sliding window 5 bp; average quality score > 20) with a minimum length of 50 bp were kept using Trimmomatic v.0.36 (Bolger *et al*., 2014). Bacterial contamination was removed from the remaining reads using DeconSeq v.0.4.3 (Schmieder & Edwards, 2011). Reads from the mock and infected samples were separately mapped against the Ec32m reference genome v.2 (Cock *et al*., 2010) using Bowtie2 v.2.2.4 (Langmead & Salzberg, 2012). Unmapped reads from the mock samples were *de novo* assembled into transcripts using Trinity v.2.4.0 (Grabherr *et al*., 2011) with default settings for k‐mer size (25 bp) and minimum contig length (200 bp). These transcripts were further used as a reference to filter out host reads from the infected samples and select only reads unambiguously assigned to *M. ectocarpii* (i.e. unmapped reads of this filtering step). Remaining reads were *de novo* assembled into transcripts using Trinity with default settings, thus constituting *M. ectocarpii* transcriptome. Read counts (i.e. gene expression) of the assembled transcripts was estimated using Rsem (Li & Dewey, 2011) included in the Trinity suite. Only transcripts with FPKM (fragments per kilobase per million reads) values > 1 were kept for downstream analysis. Completeness of the transcriptome was verified using Busco v.5.2.2 running in transcriptome mode with the eukaryote_odb10.2019‐11‐20 reference gene set (Simão *et al*., 2015). An inferred *Maullinia* proteome was generated using the longest open reading frames and the protein coding genes predicted by Transdecoder v.5.0.2 (https://github.com/TransDecoder) with default settings and used in downstream analyses. Functional annotation of the predicted genes was achieved using InterProScan v.5 (Jones *et al*., 2014).

### Additional molecular data

Transcriptome data from an Austrian population of *P. brassicae* Woronin, 1877 were taken from Ciaghi *et al*. (2018). Publicly available genomic data were taken from *P. brassicae* strains e3 (Schwelm *et al*., 2015) and PT3 (Rolfe *et al*., 2016); and *S. subterranea* (Wallr.) Lagerh. 1892 strain K13 (Ciaghi *et al*., 2018).

### *In silico* predictions of trophic mode

Busco v.5.2.2 was run in proteome mode against the eukaryote_odb10.2019‐11‐20 reference gene set (Simão *et al*., 2015) to assess completeness of all inferred proteomes, allowing for accurate predictions of trophic modes (Liu *et al*., 2021). Genomic and transcriptomic data from all three species of phytomyxean parasites in this study show a high degree of Busco completeness (< 105 missing Buscos over the total 255 Buscos in the eukaryota_odb10 database), indicating that their trophic mode can be accurately assigned by TrophicModePredictionTool. In particular, complete and fragmented Buscos amounted to: 215 for *M. ectocarpii*, 236 for *P. brassicae* e3, 233 for *P. brassicae* PT3, 194 for *P. brassicae* transcriptome and 224 for *S. subterranea* (Supporting Information Fig. S1).

The TrophicModePredictionTool tool (Burns *et al*., 2018) was used to predict the trophic mode of the investigated organisms *in silico*, based on the molecular signatures for phagotrophy, photosynthesis and prototrophy (i.e. organisms capable of synthetizing arginine, lysine, threonine, biotin, vitamin B_1_, B_2_ and B_6_; here used as a proxy for osmotrophic feeding). The code (available at https://github.com/burnsajohn/predictTrophicMode) was run in the default mode. Prediction scores enumerate the probability that an organism has the genetic toolbox to carry out the indicated function on a scale of 0–1.

Besides the three main trophic modes listed earlier, special forms of phagocytosis evolved to target a specific range of substrates, such as that of the extracellular parasite *Entamoeba histolytica* (feeding on apoptotic vertebrate cells and erythrocytes) and of the intracellular parasite *R. allomycis* (feeding on fungal cytoplasm and organelles) are predicted via an emended subset of molecular signatures of phagocytosis. Predictions were visualized as bar charts and by projecting the 4‐dimensional probability values onto a 3D tetrahedral shape representing the three trophic modes (or their absence) using scripts modified from the R package ‘pavo’ (Doucet *et al*., 2013). For static visualization of the trophic mode of an organism, the 3D tetrahedral shape with the summary prediction from each organism plotted onto it is finally rendered as a 2D circular Mollweide projection as described in Bock *et al*. (2021) and Jimenez *et al*. (2021). A detailed overview of the genes best matching the predictive molecular signatures is presented in Notes S1 for the comparison between *P. brassicae* e3 genome and *P. brassicae* transcriptome; as well as for the comparison between *P. brassicae* transcriptome and *M. ectocarpii* transcriptome.

### Fluorescent *in situ* hybridization and optical microscopy

*Plasmodiophora brassicae* was grown on the host plant *Brassica rapa* L. var. *pekinensis* (cv ‘Granat’) for 61 d before collection of root galls, thus allowing for the presence of a high number of plasmodia at different stages of development. Plants were grown at 20°C with a 12 h : 12 h photoperiod and an average irradiance of 135 μmol m^−2^ s^−1^. Galls were thoroughly rinsed in tap water to remove soil residues and preserved in Histofix 4% (phosphate‐buffered formaldehyde solution; Carl Roth, Karlsruhe, Germany) for *c*. 1 h. Following fixation, galls were dehydrated in ascending ethanol series: 10 min in 50% ethanol, twice 10 min in 70% ethanol and final storage in absolute ethanol. Galls were prepared for fluorescence *in situ* hybridisation (FISH) staining following the procedure detailed in Schwelm *et al*. (2016), with a few modifications. Briefly, galls were hand‐cut into thin sections and rinsed for 10 min in hybridization buffer (900 mM NaCl, 20 mM Tris–HCl pH 7.5, 35% formamide, 0.01% SDS) before incubation overnight at 46°C in the dark in hybridization buffer, amended with 50 ng of the FISH probe Pl_LSU_2313 (Table 1). Samples were washed twice for 20 min in washing buffer (900 mM NaCl, 20 mM Tris–HCl pH 7.5, 5 mM of NaEDTA pH 8, 0.01% SDS) at 48°C. Samples were then incubated for 20 min in Hoechst 33342 (Thermo Scientific, Waltham, ME, USA) diluted 1000× in distilled water, before being mounted in Vectashield (H‐1000; Vector Laboratories, Burlingame, CA, USA). *Maullinia ectocarpii* was grown on *E. siliculosus* Ec32m male gametophyte or *Macrocystis pyrifera* (L.) C. Agardh, 1820 CCAP1323/1 female gametophyte (same culture conditions specified earlier) for 1 month before collection. Fixation and FISH staining were achieved in the same way described for *P. brassicae*, with the following adjustments. After fixation in 4% Histofix infected algae were incubated for 2 min in 30% H_2_O_2_ to increase cell wall permeability and then dehydrated in ascending ethanol series. The hybridization was performed at 46°C overnight in the dark in hybridization buffer amended with 50 ng of probe MauJ17 (Table 1). Slides were observed with a Nikon Eclipse Ti2‐E microscope (Nikon, Tokyo, Japan) equipped with an Andor Zyla 5.5sCMOS monochrome camera (Andor Technology, Belfast, UK) and Nikon CFI Plan‐Fluor ×40/0.75 NA and ×60/0.85 NA objectives. The excitation wavelength for Hoechst 33342 was 365 nm, whereas it was 490 nm for FISH probes (Table 1). The Nis Elements software (Nikon) was used for image analysis and post‐processing (generation of overlaid images, z‐stack analysis and export of z‐stack as videos). Final figures were composed using Inkscape 0.92.4 (Inkscape Project, New York, NY, USA).

**Table 1:** Fluorescent *in situ* hybridization probes used in this study.

Probe	Organism/gene	Sequence	Dye	Excitation λ (nm)
Pl_LSU_2313	*Plasmodiophora brassicae*/28S rRNA	CCAGGCCTTTCAGCCAAGTA	6‐FAM	490
MauJ17	*Maullinia ectocarpii*/18S rRNA	CACGTCCCTCGTACCCGT	6‐FAM	490

### Transmission electron microscopy

For transmission electron microscopy (TEM), *M. ectocarpii* was grown on healthy female gametophytes of *M. pyrifera* CCAP 1323/1 in ½ strength Provasoli medium, at 10°C, under 2–6 μmol m^−2^ s^−1^ white light irradiation and 12 h : 12 h photoperiod. Biological material was chemically fixed and processed as per Murúa *et al*. (2017). Briefly, the biomass was immersed in a solution composed of 2.5% glutaraldehyde, 0.1 M cacodylate buffer at pH 7.4, 0.5% caffeine, 0.1% CaCl_2_ and 0.3% NaCl in Provasoli‐enriched seawater for 2–3 d. Post‐fixation staining was achieved with 1% OsO_4_ and 2% uranyl acetate. After dehydration in acetone series, samples were embedded in Spurr resin and polymerized at 60–70°C. Blocks were cut into 90 nm‐thick sections using an UC6 ultramicrotome (Leica, Wetzlar, Germany) mounted on copper grids and counterstained with lead citrate. Imaging was achieved with a JEM‐1400 Plus (Jeol, Akishima, Tokyo, Japan) TEM with an AMT UltraVue camera (Woburn, MA, USA). For TEM imaging of *P. brassicae*, root galls of *B. rapa* var. *pekinensis* were collected from field material in Weer (Tirol, Austria) in September 2018. Specimens were chemically fixed with 2.5% glutaraldehyde in 0.1 M cacodylate buffer containing 10% sucrose for 1 h at 4°C, rinsed with cacodylate buffer and post fixed with 1% osmium tetroxide in 0.05 M cacodylate buffer for 1 h at 4°C. After washing in cacodylate buffer, samples were dehydrated with an increasing acetone series and embedded in EMbed 812 resin. Ninety nanometre‐thick cross‐sections of root galls were cut with an Ultracut UCT (Leica), mounted on grids and counterstained with lead citrate. Sections were examined with a Libra 120 energy filter TEM (Zeiss) and images were taken with a TRS 2 × 2k high speed camera (Tröndle, Munich, Germany) and an ImageSP software (Tröndle).

## Results

### *In silico* prediction of trophic modes of Phytomyxea using genomic and transcriptomic signatures

**Fig. 1:**
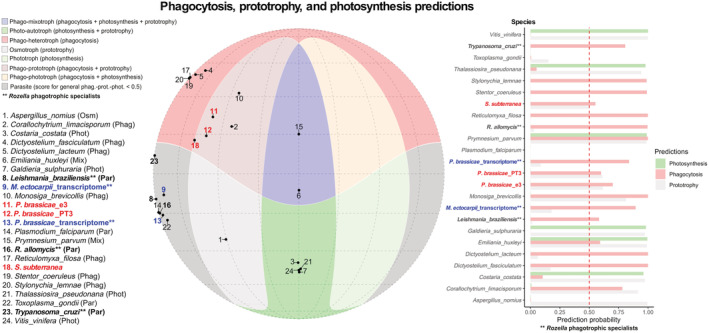
Mollweide projection showing the position of predicted phagotrophs, prototrophs and photosynthetic organisms; and bar chart showing the scores of individual prediction probabilities for the same organisms. Colored regions (red, blue, beige, green) indicate overlapping areas where individual predictions were > 0.50. Dark‐grey shaded regions indicate areas where all three predictions were < 0.50. Note that phagotrophic specialists (such as *Rozella*‐like phagotrophs) do not map the three main trophic categories and fall in the grey area due to the Mollweide projection only using the general phagocytosis prediction for each organism. Each numbered black dot indicates one of the 19 organisms used as a reference to test the model. The same organisms are represented in the bar chart where prediction probability scores are shown as coloured bars (green, photosynthetic; grey, prototroph; brown, phagotroph). The 0.5 threshold, above which a prediction is deemed valid, is indicated by the red dashed line. Names in red/bold indicate the phytomyxid genomes tested in this study while blue/bold indicates phytomyxid transcriptomes; names in bold and followed by double asterisks (**) indicate organisms for which the strongest prediction is *Rozella*‐type intracellular phagotrophy.

All analysed phytomyxids datasets bear molecular signatures of phagotrophy (Fig. 1; Table S1). *Plasmodiophora brassicae* (e3 and PT3) and *S. subterranea* (SSUBK13) genomes score high for phago‐prototrophy (red nos. 11, 12 and 18 on the Mollweide projection in Fig. 1). The prediction scores from the genome data in *P. brassicae* are *c*. 60% for prototrophy (e3 = 0.615 and PT3 = 0.612) and are similar for general phagotrophy (e3 = 0.700; PT3 = 0.600). The prototrophy score for *S. subterranea* is lower (SSUBK13 = 0.500, bar chart in Fig. 1), as is the score for general phagotrophy (SSUBK13 = 0.552). When the subset of signatures predicting *Rozella*‐like intracellular phagotrophy is considered the probability scores increase to nearly 100% for the genome datasets (e3 = 0.978; PT3 = 0.983 and SSUBK13 = 0.967; bar chart in Fig. 1). The probabilities for photosynthesis and entamoebid‐like phagotrophy (a second peculiar mode of phagotrophy mostly observed in extracellular endoparasites such as *Entamoeba*) remain below the threshold of 50% in the genomic data (Table S1). When the proteomes inferred from the transcriptomes of *P. brassicae* and *M. ectocarpii* are tested the score for general phagotrophy and prototrophic predictions are very low (< 0.21). The *Rozella*‐like phagotrophy remains high with a score of 0.838 in *P. brassicae* and 0.894 in *M. ectocarpii* (bar chart in Fig. 1). The transcriptome datasets are placed in the section ‘Parasite’ of the Mollweide projection and map close to the intracellular fungal parasite *R. allomycis* (red nos. 9 and 13 and black no. 16 in the Mollweide projection in Fig. 1), while the genomic datasets are in the phago‐prototroph area. The assignment to the ‘Parasite’ area in the Mollweide projection highlights a low score (< 0.5) for the main trophic categories (i.e. general phagotrophy, prototrophy and photosynthesis), but does not exclude the assignment to specialized sub‐categories of phagocytosis (i.e. *Entamoeba* or *Rozella*‐like phagocytosis) as highlighted by the bar chart in Fig. 1.

In *P. brassicae* (for which both genomic and transcriptomic data are available), a detailed look at the molecular signatures highlighted that nearly half (14/29) of the phagotrophy‐related genes driving the genome apart from the transcriptome were associated with cilia/flagella (as per their GO term annotation, Notes S1). Within the predictive model, flagella and cilia are describers of the phago‐prototrophic niche, which accommodates organisms using these structures to feed (e.g. choanoflagellates such as *Monosiga brevicollis*; Fig. 1).

On the other hand, trophic predictions for the transcriptomic datasets of *P. brassicae* and *M. ectocarpii* were similar (Fig. 1). In *P. brassicae* genes associated with phagotrophic signatures in the transcriptome were linked to GO terms involving the cytoskeleton (14/40), the cytosol (6/40) and the mTOR complexes (5/40), including the GO terms TORC2 complex, Seh1‐associated complex and the lysosome gene RRAGA (Notes S1). A closer look at the predicted functions highlights their potential involvement in processes such as signal transduction, cell reorganization/polarization, metabolism and cell cycle. In particular, Ras GTPases, mTORC1 and mTORC2 complexes are strong descriptors of *Rozella*‐like phagotrophic behaviour and describe nearly half (10/21) of the signatures shared between the transcriptomes of *P. brassicae* and *M. ectocarpii* (Notes S1).

### Microscopic evidence of phagocytosis in intracellular plasmodia of *P. brassicae* and *M. ectocarpii*

**Fig. 2:**
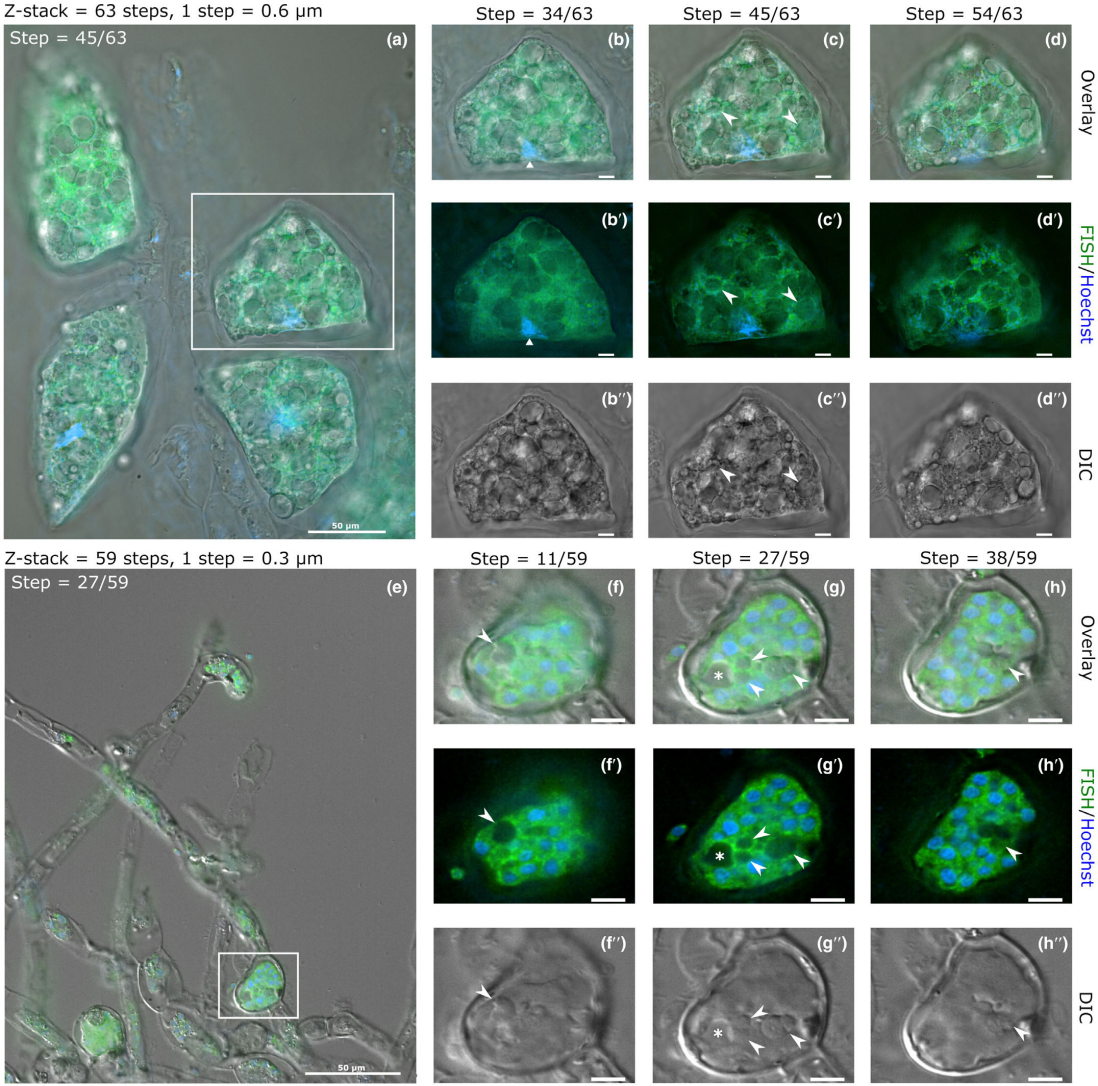
Optical and fluorescence micrographs provide evidence of the internalization of host organelles in intracellular plasmodia of *Plasmodiophora brassicae* in *Brassica rap*a var. *pekinensis* (a–d) and of *Maullinia ectocarpii* in *Ectocarpus siliculosus* Ec32m (e–h). Images have been captured using differential interference contrast microscopy (b″–d″, f″–h″) and fluorescence microscopy (b′–d′, f′–h′) and subsequently overlaid (a–d, e–h). Fluorescence *in situ* hybridisation (FISH) probes specific to the 28S rRNA gene of *P. brassicae* and 18S rRNA gene of *M. ectocarpii* were used to highlight the ribosome‐rich cytoplasm of the parasites (green). In fluorescence microscopy, Hoechst staining highlighted in blue the nuclei of both the parasite (small and numerous) and its host (single big nucleus in the plant host (a–d); not visible in the algal host (e–h)). White squares in (a, e) indicate the plasmodia shown in detail in (b–d) and (f–h), respectively. The white triangle in (b) points towards the Hoechst‐stained host cell nucleus, while white arrowheads in (c) indicate two completely internalized starch granules. Arrowheads in (f–h) highlight engulfed algal phaeoplasts, while the asterisk in (g) indicates a vacuole. Focal planes represent a high (b, b′, b″, f, f′, f″), a central (c, c′, c″, g, g′, g″) and a low (d, d′, d″, h, h′, h″) layer from z‐stacks containing entire plasmodia. Bars: (b–d) 10 μm; (f–h) 5 μm.

Microscopic observation of intracellular biotrophic plasmodia of *P. brassicae* and *M. ectocarpii* support phagotrophy of host organelles by the parasites. Mature feeding plasmodia of *P. brassicae* (Fig. 2a) could be recognized by the high number of small nuclei (Fig. 2b′–d′, small blue dots) in the absence of cytoplasm cleavage. The plant nucleus was still present and could be distinguished from the parasite nuclei by its larger dimensions (Fig. 2b,b′; white triangle). Plasmodia filled up the host cells entirely (Fig. 2a–d; green), leaving little free space within the cell wall. Abundant starch grains were easily identified in differential interference contrast microscopy by their shape, size, hyaline texture and tridimensional appearance (Fig. 2b″–d″). A high number of starch grains was located between the plant cell wall and the parasite plasma membrane, pressed against the plasmodium as if superficially ‘plugged’ in membrane pockets (Fig. 2b,b′,d,d′). Many starch grains were also found to be completely enveloped by the parasite plasmodium, often contiguous to other engulfed and ‘plugged’ starch grains, giving the plasmodium an overall ‘sponge‐like’ or ‘trabecular’ aspect in fluorescence microscopy (Fig. 2b′–d′). Two starch grains were entirely surrounded by the plasmodium (Fig. 2c,c′,c″, white arrowheads; videos in Notes S2) as highlighted by the presence of green hue around them and in the focal planes above and below them.

*Ectocarpus siliculosus* cells infected by mature *M. ectocarpii* were easily distinguishable thanks to the clear signs of hypertrophy (Fig. 2e, white square). The plasmodium shown (Fig. 2f–h) occupied the majority of the space within the host cell wall, as indicated by the green FISH staining of its cytoplasm. The plasmodium was multinucleated (Fig. 2f–h,f′–h′, blue signal) and showed vacuolar structures where no green fluorescence could be observed (Fig. 2f–h,f′–h′, white arrowheads and asterisk; videos in Notes S2). Some vacuoles contained refractive structures consistent with the phaeoplasts of *E. siliculosus* (Fig. 2g,g′,g″, white arrowheads) while other did not (Fig. 2g,g′,g″, white asterisk). Phaeoplasts were also observed to be ‘plugged’ in membrane pockets (Fig. 2f,f′,f″,h,h′,h″, white arrowhead), much like starch grains in *P. brassicae*. Scans of the entire volume of the investigated plasmodia along the *z*‐axis are available as videos (Notes S2); allowing for a better visualization of the host organelles engulfed by the parasites. To further strengthen our observations, we performed a FISH experiment on *M. ectocarpii* infecting the female gametophyte of the kelp *M. pyrifera*. Even in this case, phagocytosis was observed as highlighted by the observation of phagocytic vacuoles and the late phagocytosis of the host nucleus (Fig. S2; Notes S3).

### Ultrastructural evidence of phagocytosis in intracellular plasmodia of *P. brassicae* and *M. ectocarpii*

**Fig. 3:**
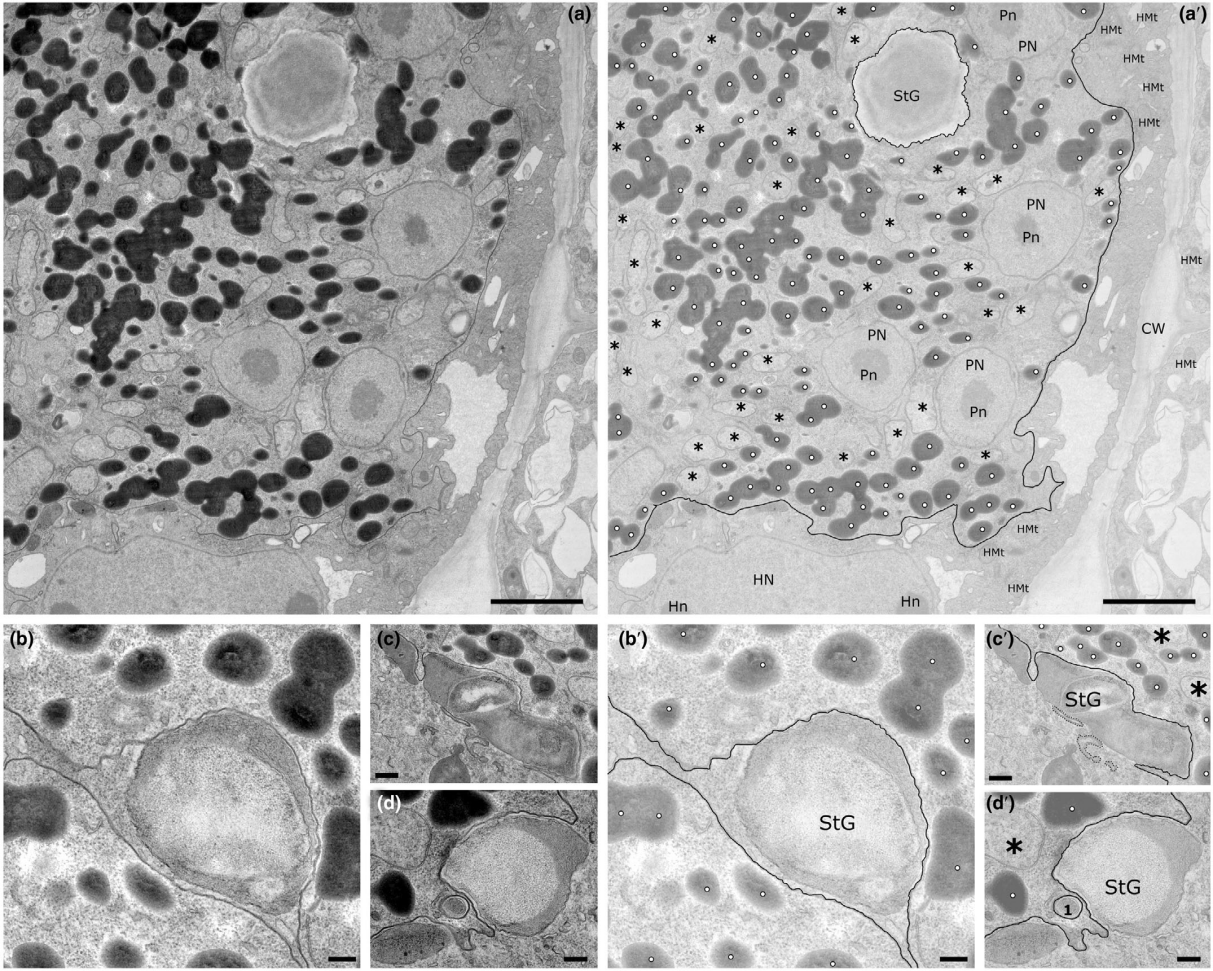
Plasmodia of *Plasmodiophora brassicae* growing within the cortical cells of the root of *Brassica rapa* ssp. *pekinensis*. Annotations are provided in a separate image from the original picture (e.g. annotations for a in a′, etc.). (a, a′) Overview of the interface between the plasmodium and its host. Note the starch granule surrounded by the plasmodium. (b, b′) Starch grain from desegregated amyloplasts is surrounded by two plasmodial protrusions, one of which in closely appressed to the granule surfaces. (c, c′) Detail of plasmodial pseudopodium‐like processes encircling a starch grain. (d, d′) Plasma membrane invagination partially surrounding a starch grain. Note a small phagosome containing a membrane‐bound, electron‐opaque unidentified organelles (dashed line). In all pictures, note the presence/absence of lipid droplets and the different electron opacity/organization of the mitochondria, used as main distinctive features to tell apart host and parasite. CW, host cell wall; HMt, host mitochondria; Hn, host nucleolus; HN, host nucleus; Pn, parasite nucleolus; PN, parasite nucleus; StG, starch grains; white dots, parasite lipid droplets; *, parasite mitochondria; black lines indicate the plasma membrane of the plasmodium; solid line, plasma membrane; dotted line in (c, c′), putative plasma membrane; no. 1 in (d, d′), putative phagosome. Bars: (a, a′) 2500 nm; (b, b′, c, c′) 500 nm; (d, d′) 250 nm.

Plasmodia and thick‐walled resting spores of *P. brassicae* were observed inside the cortical cells of *B. rapa* ssp. *pekinensis*. Plasmodia can be discriminated from the plant host because of the high amount of lipid globules stored within the cytoplasm (absent from healthy plant cortical cells), the different electron opacity of the cytoplasm and the relatively poorly stained mitochondria with sparse tubular cristae (Fig. 3). Multinucleate plasmodia occupy most of the host cell, leaving space only for the host nucleus, small vacuoles and few smaller organelles (like mitochondria) embedded in a film of plant cytoplasm appressed to the cell wall. Parasite nuclei were clearly distinguishable from the plant nuclei, because of their rounder shape and smaller size (Fig. 3a,a′). The overall shape of the plasmodium was irregularly lobed, to the extent that often it was impossible to clarify whether a single highly lobed or many different plasmodia were inhabiting the same host cell (Fig. 3a,a′). Lobes of different shape and size were often found surrounding and/or closely appressed to starch grains (Fig. 3b,b′), originated from desegregated amyloplasts. Often pseudopodia‐like processes and membrane invaginations seemed to encircle and close around starch grains (Fig. 3c,c′,d,d′) and in one occasion one of those granules was found to be completely surrounded by the plasmodium (Fig. 3a, StG). The mitochondria of *P. brassicae* were found to be generally electron‐translucent and contained fewer cristae than the lamellar plant mitochondria (Fig. 3c,c′,d,d′, asterisks). Mitochondria in thick‐walled resting spores were much better defined in their ultrastructure and are overall more electron opaque (Fig. S3).

**Fig. 4:**
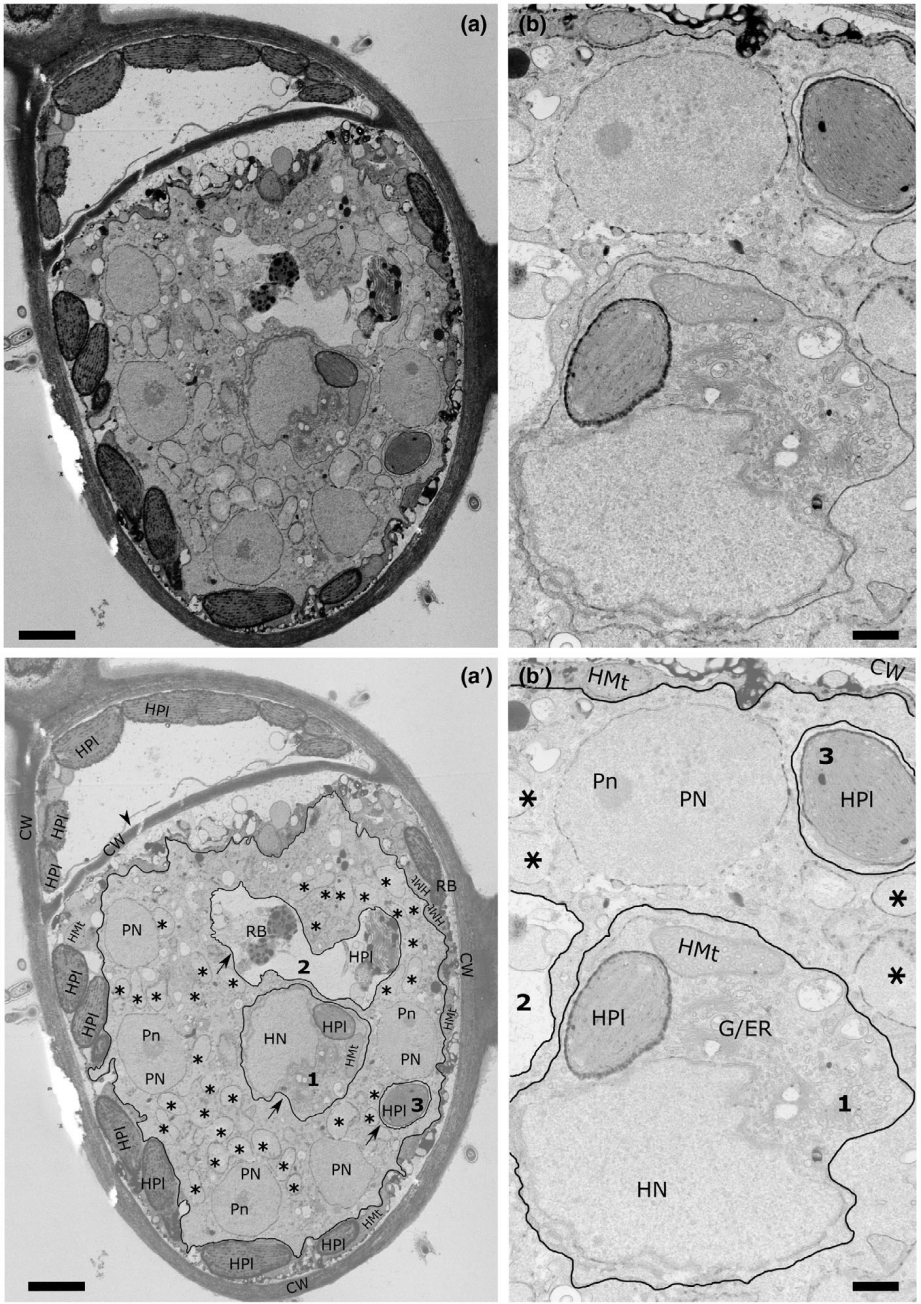
Plasmodium of *Maullinia ectocarpii* growing within a cell of the gametophyte of *Macrocystis pyrifera*. Annotations are provided in a separate image from the original picture (e.g. annotations for a in a′). (a, a′) Overview of the interface between the plasmodium and its host. Note the three vacuoles containing algal mitochondria, phaeoplasts and nucleus bound by membrane and surrounded by the parasite plasmodium (nos. 1–3, arrows). (b, b′) Close up of (a, a′) highlighting details of the algal organelles surrounded by the parasitic plasmodium. Note the difference in electron opacity/granularity between the parasite and host cytoplasm used as main distinctive features to tell apart host and parasite. Secondarily, note the absence of clear tubular cristae in the putative mitochondria of *M. ectocarpii*. CW, host cell wall; HMt, host mitochondria; Hn, host nucleolus; HN, host nucleus; HPl, host phaeoplasts; Pn, parasite nucleolus; PN, parasite nucleus; RB, residual bodies; *, putative parasite mitochondria; black lines indicate the plasma membrane of the plasmodium; arrows and numbers, putative phagocytic vacuoles; arrowhead, cell wall separating infected and non‐infected sectors within the same cell. Bars: (a, a′) 2000 nm; (b, b′) 500 nm.

Feeding plasmodia of *M. ectocarpii* were observed in intercalary and tip cells of the filamentous female gametophytes of *M. pyrifera* (Fig. 4). Plasmodia readily occupy the whole host cell, initially taking up the space of the central vacuole, thereby pushing the organelles towards the periphery of the cell. *M. ectocarpii* plasmodia are easily discriminated from the host cell by the absence of phaeoplasts and because of the difference in the cytoplasmic electron opacity (i.e. opaquer in the alga; Fig. 4). Electron dense mitochondria with tubular cristae have been noticed in the algal host. In the plasmodia of *M. ectocarpii*, mitochondria are not as visible: putative mitochondria appear as double‐membrane bounded electron translucent structures without clearly discernible tubular cristae (Figs 4a,a′,b,b′, asterisks, S3). Comparably with observations in *P. brassicae*, in mature *M. ectocarpii* plasmodia/zoosporangia, mitochondria within zoospores show a higher level of structure, being electron‐denser and with well‐organized tubular cristae (Fig. S3). Plasmodia are irregular and sometimes structures similar to pseudopodia can be observed, especially in very young, developing plasmodia which do not yet fill the host cell (Fig. S4). Vacuoles are often observed within *M. ectocarpii* plasmodia and differ in size and content (Fig. 4a′,b′, arrows and nos. 1–3). Vacuoles can be nearly empty (electron translucent), but most vacuoles are either loosely filled with degraded material (Fig. 4a′,b′, no. 2) or filled with host organelles and cytoplasm (Fig. 4a′,b′, nos. 1 and 3). In Fig. 4(b,b′) vacuole no. 3 can be observed containing a phaeoplast, with little to no space for other structures. A second, bigger vacuole (no. 1) contains the host nucleus together with a phaeoplast, one host mitochondrion and host cytoplasm, in turn containing membranous structures interpreted as endoplasmic reticulum and/or Golgi apparatus. An even bigger vacuole (no. 2) can be observed in Fig. 4(a,a′), within which a clearly degraded phaeoplast and two residual bodies, potentially representing a further stage in phaeoplast degradation, can be observed. The presence of a degraded phaeoplast in vacuole 2 suggests that this has been isolated from the rest of the host cytosol and digested. Presumably, vacuoles 1 and 3 are bound to undergo the same process. The plasmodium itself is multinucleate but it has not yet undergone cytodieresis and zoospore cleavage.

## Discussion

In this study, by analysing complementary lines of evidence, we demonstrate that phagocytosis is a trait that phytomyxean parasites conserved from free‐living Rhizarian ancestors, adapting it to the intracellular environment where it underpins the biotrophic interaction and where it coexists with specialized strategies of host manipulation. Molecular signatures of phagocytosis are present in all phytomyxean datasets analysed; but the model aggregates datasets in different trophic modes according to genome‐based and transcriptome‐based signatures (Fig. 1). These different predictions can be explained in the light of the polyphasic phytomyxean life cycle (Liu *et al*., 2020), where the transcriptome provides a realized molecular snapshot of the feeding stage while the genome also contains genetic information on stages other than the intracellular plasmodium (e.g. free‐living flagellated zoospores). Genomic signatures identify Phytomyxea as phago‐prototrophs while transcriptomes of intracellular parasitic stages are best explained by the subset of signatures of the intracellular phagotrophic specialist *R. allomycis* (Powell *et al*., 2017; Fig. 1). Molecular signatures associated with the flagellum are the main drivers assigning *P. brassicae* genomes to the phago‐prototrophic niche. While flagella are associated with phagotrophy in certain organisms (e.g. choanoflagellates); in Phytomyxea, flagella are exclusively associated with locomotion in the zoosporic phase of the life cycle but play no role in nutrition (Barr & Allan, 1982; Parodi *et al*., 2010; Feng *et al*., 2012). On the other hand, molecular signatures belonging to the Ras GTPases, mTORC1 and mTORC2 complexes are shared between *P. brassicae* and *M. ectocarpii* transcriptomes, both assigned to the *Rozella*‐like phagotrophic specialists. Ras GTPases are known to control cytoskeletal remodelling and vesicular trafficking in human phagocytes (Wiedemann *et al*., 2005) and mTORC2 has been linked to cytoskeletal polarization related to budding in yeasts (Loewith *et al*., 2002). Furthermore, mTOR complexes, particularly mTORC1, are known to be paramount sensors of the nutritional state of the cell acting as a switch between anabolic and catabolic metabolism; and more broadly between growth and proliferation on one hand and autophagy and apoptosis on the other (Sabatini, 2017; Condon & Sabatini, 2019). This hints at a pivotal role of perception of the nutritional state and signal transduction in the intracellular feeding plasmodia of phytomyxids, coupled with cytoskeletal rearrangements that are central for phagocytic behaviour.

Molecular evidence shows that phytomyxean plasmodia rely on a reduced molecular machinery to perform intracellular phagotrophy, similarly to what happens in the intracellular fungal parasite *R. allomycis* (Burns *et al*., 2018). *Rozella allomycis* belongs to the Cryptomycota, an early‐diverged group within the true Fungi (James *et al*., 2013) which has been demonstrated to be capable of intracellular phagocytosis (Powell *et al*., 2017). Ultrastructural and molecular data agree in showing that *R. allomycis* mitochondria are hypo‐functional in intracellular phases of the life cycle and that the parasite relies on the host ones to source ATP (James *et al*., 2013; Powell *et al*., 2017), thus hinting at a trophic and energetic reliance on the host. Despite the wide phylogenetic distance separating Cryptomycota and Phytomyxea, molecular signatures of phagotrophy from *Rozella* seem to accurately describe the behaviour of intracellular plasmodia in Phytomyxea.

Trophic dependency in intracellular plasmodia of Phytomyxea is also supported by ultrastructural observations of *P. brassicae* and *M. ectocarpii*, highlighting big, electron translucent and nearly featureless mitochondria (Fig. S3). Mitochondria have been proven to be highly adaptable and plastic organelles in intracellular parasites with complex life cycles, where mitochondrial shape and structure readily mirror changes in metabolic strategies, in turn driven by host or life‐phase switches (Voleman & Dolezǎl, 2019; and references therein). The smaller and denser mitochondria with defined cristae observed in phytomyxean spores (Fig. S3; Talley *et al*., 1978) and the already reported co‐presence of microbodies in zoospores (Tanaka *et al*., 2001) is consistent with the usage of stored lipids as primary energy source (Held, 1975; Powell *et al*., 2017). Taken altogether, this evidence suggests a metabolic switch from complete reliance on the host during the intracellular growth, to zoosporic reliance on stored fatty acids in Phytomyxea and relates it to distinct changes in mitochondrial activity. Indeed, in contrast to *Rozella*, analyses of the mitochondrial genome of *P. brassicae* did not highlight any particular lack in functional genes (Daval *et al*., 2018; Stjelja *et al*., 2019), suggesting that the mitochondrion is still completely functional in other parts of the life cycle.

Phagocytosis in Phytomyxea has adapted to their intracellular lifestyle. Feeding plasmodia appear to uptake host structures by protrusion of lobes and invagination of the plasma membrane, again reminiscent of processes observed in *R. allomycis* (Powell *et al*., 2017), but also akin to the so‐called ‘prey infiltration’ strategy used by phylogenetically related free‐living amoebae in the order Vampyrellida (Hess & Suthaus, 2022), that, however, remain outside the host cell membrane. Our observations in TEM and fluorescent microscopy show different host organelles engulfed by *M. ectocarpii*, although phagocytosis seem skewed towards phaeoplasts (Figs 2, 4). Likewise, *P. brassicae* seems to target preferentially the host amyloplasts (Figs 3, 4). Whether this seemingly selective uptake of host organelles is the outcome of real targeting, of chance (phaeoplasts and amyloplasts are the most widespread organelles in the respective host cells) or an observational artefact caused by the delayed digestion of complex plastid‐derived organelles remain to be ascertained.

However, it is known that host manipulation by *P. brassicae*, beside inducing mitosis and cellular expansion in the host (Olszak *et al*., 2019); generates a strong physiological sink, driving photosynthates to the infected host cells (Malinowski *et al*., 2019). Those photosynthates accumulate as starch in amyloplasts, which are significantly more abundant in the infected root cells of brassicas (Ma *et al*., 2022) where they appear to be superficially ‘plugged’ into the plasmodium surface, reminiscent of a process of semi‐extracellular phagocytosis (named ‘pomacytosis’, Kamennaya *et al*., 2018). Previous studies highlighted an upregulation of the plastidial MEX1 maltose transporter in infected roots, involved in the export of maltose outside the plastid after starch degradation (Badstöber *et al*., 2020; Fig. S5). In this context, we can hypothesize that the pomacytosis‐like process observed in *P. brassicae* co‐opts phagocytosis and generates a close interface between the parasite and the amyloplast, allowing the consumption of leaked sugars without cutting the organelle off from the nucleus. This, in turn, allows for a constant supply of newly synthesized starch in the semi‐engulfed amyloplasts by maintaining essential plastid‐to‐nucleus retrograde signalling (Enami *et al*., 2011).

Recently identified *P. brassicae* glucose transporters and glucose content in infected roots have been found to significantly increase in late stages of infection (Kong *et al*., 2022). In the context of a biotrophic interaction that relies on phagotrophy, feeding specifically on amyloplasts has the clear advantage of targeting the host's carbon storage while avoiding organelles needed for the host cell survival and regulation (e.g. the nucleus). This, in turn, keeps intact the molecular machinery necessary for the host cell to continue accumulating photosynthetate as amyloplastic starch, giving time to the parasite to complete its life cycle. Nonetheless, it is unclear whether the targeted phagocytosis/pomacytosis of amyloplasts is an active process or one passively driven by space‐constraints within host cells packed with energy‐rich organelles (Figs 3, 4). Further evidence needs to be produced to confirm this hypothesis; but if confirmed, this would place *P. brassicae* in a particular ecological niche where ancestral phagocytosis provided the baseline to exploit host resources obtained via molecular manipulation co‐evolved with specific hosts (Pérez‐López *et al*., 2020, 2021; Hossain *et al*., 2021).

Results gathered from *M. ectocarpii* are coherent with the trophic mode observed for the rest of the Phagomyxida, especially with *P. algarum*, an intracellular phagotrophic parasite of brown algae and the first described species within this taxon (Karling, 1944). Furthermore, these findings also support intracellular phagocytosis as the main mode of nutrition within Phytomyxea. More so since, differently from *P. brassicae* data derived from sporogenic plasmodia, molecular and morphological data for *M. ectocarpii* come from sporangial plasmodia (Maier *et al*., 2000). As per previous reports (Maier *et al*., 2000), it is interesting to notice that the *M. ectocarpii* plasma membrane is thinner than that surrounding *P. brassicae* (Williams & McNabola, 1970). Whether this difference in host–parasite interfaces is a species‐specific apomorphy, a life stage‐driven feature, or whether and how it influences the differences observed in intracellular phagocytosis between the two parasites (e.g. more frequent ‘pomacytosis’ in *P. brassicae*) remains to be tested. Nonetheless, following a conservative and parsimonious interpretation, our results hint at a key role of intracellular phagocytosis in both the sporangial and sporogenic phases of the phytomyxean life cycle. *Maullinia ectocarpii* also induces mitosis and cell expansion in its algal host (Maier *et al*., 2000) but evidence on carbohydrate accumulation in infected tissue has not yet been produced. Brown algae accumulates photosynthates mainly as soluble vacuolar laminaran and cytoplasmic mannitol (Michel *et al*., 2010; Chabi *et al*., 2021). It is therefore interesting to notice the early disappearance of the vacuole in cells infected by *M. ectocarpii*. Although consumption of the vacuole seems to be a necessary step of intracellular colonization, simply to provide growth space for the enlarging sporangium; this would also allow for the parasite to immediately consume the major polysaccharide storage within the host cell providing it with rapid energy. However, a first glance at levels of gene expression in *Maullinia*‐infected *E. siliculosus* Ec32m did not highlight a clear pattern of upregulation of laminarin/mannitol catabolism or extra‐vacuolar transport of carbohydrates (Table S2), thus whether *M. ectocarpii* manipulates its host cell carbohydrate metabolism remains unclear. It is worth reminding that the transcriptome analysed here originated from an asynchronous parasite population, where the signal of a possibly transient and/or very local interaction between the parasite and the host vacuole could have been diluted in the bulk approach used.

In the case of *M. ectocarpii*, phaeoplasts seemed to be a preferred target of phagocytosis. In fact, TEM images show that phaeoplasts in infected cells shrink with the progression of the infection (Figs 4, S4). In‐depth investigation of plastidial dynamics in infected algal cells is beyond the scope of this study, but the possibility that plastids are directly manipulated before being targeted for consumption by the parasite cannot be excluded. If proven, this would hint at a conservation or convergence of the target host organelle within the Phytomyxida. Similar patterns of plastidial shrinkage have been highlighted in the interaction between the intracellular oomycete parasite *Anisolpidium ectocarpii* (infecting *M. pyrifera*), but in this case the decrease in size was interpreted as a result of autophagic processes and thus to the reaction of the host against the parasite (Murúa *et al*., 2020).

It is worth bearing in mind that only scarce information is available on the sporogenic stage of *M. ectocarpii* (Parodi *et al*., 2010; Blake *et al*., 2017) and on Phagomyxida overall (Schnepf *et al*., 2000; Murúa *et al*., 2017). If, as it is suspected, a sporogenic phase inducing gall formation in adult kelp sporophytes does exist (Blake *et al*., 2017), an even higher degree of host manipulation can be expected for *M. ectocarpii*, bringing it even closer to its land‐dwelling relative *P. brassicae*.

Although not common, intracellular endocytosis has been documented in intracellular parasites spanning the taxa Apicomplexa (Spielmann *et al*., 2020; and references therein), Cryptomycota (Torruella *et al*., 2018) and Euglenozoa (Etheridge, 2022). The reduced dataset of phagotrophy‐related proteins from *R. allomycis* correctly describes *T. cruzii* and *Leishmania braziliensis* as capable of intracellular phagotrophy (Chasen *et al*., 2020; Halliday *et al*., 2020) while fails to assign *Plasmodium falciparum* and *T. gondii* to this category. Indeed, *P. falciparum* and *T. gondii* are known to use a different set of genes to undertake endocytic nutrient uptake (Spielmann *et al*., 2020) and especially to lack important genes involved in small GTPase (RAS superfamily) and TOR signalling pathways (Van Dam *et al*., 2011), which are fundamental for the predictive model (Burns *et al*., 2018). The apparent proximity of the genetic make‐up underpinning intracellular phagocytosis in unrelated Phytomyxea, *Rozella* and trypanosomatids is intriguing, since it hints at the possibility that the smallest subset of genes required for phagocytosis is present in these otherwise unrelated parasites. Genome reduction is a well‐known process in intracellular parasites (Keeling & Slamovits, 2005) and those among them which maintain a phagocytic behaviour might make a good model to investigate the very core of the phagocytic machinery, when not overly specialized towards their host or a specific substrate.

The data presented and discussed here place phytomyxean intracellular parasites half‐way between the extremes of specialized biotrophic host manipulation and osmotrophy, and generalist phagocytic predation. Growing molecular and microscopic evidence suggests that phagocytosis is a backbone feature of Rhizarians upon which ‘variations on the theme’ brought about the diversification of the group (Anderson, 1978; Hirakawa, 2017; Gerbracht *et al*., 2022; Hess & Suthaus, 2022). In this context, Phytomyxean are not an exception. It is tempting to speculate that the maintenance and adaptation of phagocytic behaviour is one of the reasons behind the success of this impactful and recalcitrant parasites, allowing them to specialize to certain hosts meanwhile maintaining the ability to feed and propagate within a broader set of organisms (Ludwig‐Müller *et al*., 1999; Maier *et al*., 2000; Qu & Christ, 2006). Further research on this group of intriguing parasites will surely provide more evidence on the degree of host manipulation/phagocytosis within the class, especially if targeted towards non‐model organisms for which data are lacking. Comparative investigations and the exploration of biodiversity surrounding parasites and pathogens prove paramount to deeply understand their biology and potentially devise strategies to counter their effects and broadly foresee the evolutionary trajectories of parasitism.

## Competing interests

None declared.

## Author contributions

AG and SN planned and designed the research. AG, MH, PM, WS and MK generated microscopic images and analyses. CMMG, SN, SC prepared and collected samples for molecular analyses. AG, JAB, SC, SLH provided bioinformatics analyses and pipelines. AG and SN wrote the first draft of the manuscript which was finalized with the input of all authors.

## Supporting information


**Fig. S1** Busco analysis of the genomes and transcriptomes of all Phytomyxid analysed.
**Fig. S2** Optical and fluorescence micrographs provide evidence of phagocytosis in intracellular plasmodia of *Maullinia ectocarpii* in *Macrocystis pyrifera*.
**Fig. S3** Young plasmodium of *Maullinia ectocarpii* in its host alga *Macrocystis pyrifera*.
**Fig. S4** Comparison of TEM images of mitochondria of different phytomyxea and life cycle stages.
**Fig. S5** Changes in *Brassica oleracea* starch metabolism during infection from *Plasmodiophora brassicae*.

**Notes S1** Comparison between best‐hit genes against molecular signatures of general phagotrophy.

**Notes S2** Accompanying video for Fig. 2.
**Notes S3** Accompanying video for Fig. S2.

**Table S1** Values from the TrophicModePredictionTool assigning the proteomes of the listed organisms to five different trophic modes.

**Table S2** Log_2_ fold change expression levels in *Maullinia*‐infected *Ectocarpus siliculosus* Ec32m vs non‐infected control for enzymes involved in carbohydrate metabolism.
Please note: Wiley is not responsible for the content or functionality of any Supporting Information supplied by the authors. Any queries (other than missing material) should be directed to the *New Phytologist* Central Office.

## Data Availability

Raw NGS sequences for the transcriptomic dataset of *M. ectocarpii* and its host *E. siliculosus* have been deposited in NCBI Sequence Read Archive (SRA) under the BioProject accession no. PRJNA878940. Publicly available datasets used in this work are referenced in detail by the relative publications.
